# Ultrasonography measurement of glottic transverse diameter and subglottic diameter to predict endotracheal tube size in children: a prospective cohort study

**DOI:** 10.1038/s41598-022-19668-6

**Published:** 2022-09-08

**Authors:** Chanya Deekiatphaiboon, Maliwan Oofuvong, Orarat Karnjanawanichkul, Sirikarn Siripruekpong, Pattamawan Bussadee

**Affiliations:** Department of Anesthesiology, Faculty of Medicine, Prince of Songkla University, 15 Kanjanavanich Road, Hat Yai, Songkhla, 90110 Thailand

**Keywords:** Anatomy, Medical research

## Abstract

We aimed to determine the correlation between mid-glottic transverse diameter/subglottic diameter and outer diameter of endotracheal tube (ETT) by ultrasonography in children. Ninety-five patients aged 1–8 years who underwent general anesthesia were included. Ultrasonography of glottic/subglottic transverse diameter was performed by two investigators after patients were anesthetized and when the train of four showed ≤ 4. The subglottic diameter was measured at the mid cricoid cartilage. The mid-glottic transverse diameter was measured at the mid-point of true vocal fold triangle whereas the distance between arytenoids was considered as the glottic transverse diameter. Linear regression models and correlation coefficients (r) were used to determine the best formula of glottic/subglottic transverse diameter to predict the outer diameter of ETT. The predicted outer diameter of ETT formula for subglottic diameter, mid-glottic transverse diameter, and glottic transverse diameter were 5.7 + (subglottic_mm_/3) with an r of 0.45, 5.5 + (midglottic_mm_/2) with an r of 0.47, and 5.7 + (glottic_mm_/4) with an r of 0.46, respectively. The correlation between subglottic diameter and mid-glottic transverse diameter was 0.50. Subglottic/mid-glottic/glottic transverse diameter formulae had moderate correlations with the outer diameter of ETT. The glottic/mid-glottic transverse diameter can be used alternatively to predict the ETT size.

Trial registration: Thai Clinical Trial Registry: TCTR20191022002 Registered 22/10/2019—Prospectively registered, https://www.thaiclinicaltrials.org/# TCTR20191022002.

## Introduction

Intubation is the definitive method of airway management. Inappropriate endotracheal tube (ETT) size, especially in children, may result in poor ventilation, unreliable end-tidal gas monitoring, and airway damage. Selecting the appropriate ETT size is especially important to prevent complications of intubation. Predictive formulae for appropriate ETT size in children have been based on age, weight, height, and the radius of the little finger^1–5^. Age-based formulae are the simplest for physicians compared with the others. However, the correlation of age-based formulae for pediatric ETT size selection by the Cole formula was as low as 47–77% in previous studies^2,5,6^. Using age-based formulae tends to predict ETT sizes that are too small^7^. The diameter of the subglottic upper airway measured by ultrasonography in pediatric patients has been shown to have good correlation with ETT size^6–9^. Nevertheless, the transverse diameter at the level of the vocal cord being the narrowest portion of the larynx in children, compared to the cricoid ring, has been mentioned in studies of magnetic resonance imaging (MRI)^10^ and rigid video-bronchoscopy^11^. Based on the anatomy of the glottis described by Eckel et al.^12^, the transverse plane of the glottis has two different parts in the vertical axis, separated by the tip of the vocal process. There are ligamental parts and arytenoid parts of the glottis^12,13^. In a spontaneous breathing patient, the ligamental part, which we considered as the mid-glottic transverse diameter, might be the narrowest portion in the glottic transverse plane compared to the cricoid ring^13^.

To date, whether mid-glottic transverse diameter is the narrowest part in the child's larynx compared to the cricoid ring is still controversial. Moreover, use of the mid-glottic transverse diameter by ultrasonography to predict ETT size in children has never been determined. Therefore, we aimed to predict the outer diameter (OD) of ETT in children undergoing general anesthesia using the mid-glottic transverse diameter and subglottic diameter based on ultrasonography as well as to compare the relationship between the mid-glottic transverse diameter and subglottic diameter.

## Methods

This prospective observational study was approved by the Human Research Ethic Committee, Faculty of Medicine, Prince of Songkla University (REC. 62-133-8-4). We confirmed that all methods were performed in accordance with the relevant guidelines and regulations. The study was registered in the Thai Clinical Trial Registry (www.thaiclinicaltrials.org) on 22/10/2019 (TCTR ID: TCTR20191022002). We enrolled children at the age of 1–8 years, American Society of Anesthesiologists (ASA) classification I–III patients, who received general anesthesia with an endotracheal tube between January and August 2020. Exclusion criteria included (1) pre-existing diagnoses or suspected laryngeal or tracheal pathology, (2) anticipated difficult mask ventilation and difficult intubation, (3) presented risk of aspiration that required rapid sequence induction, and (4) the child's parents or guardians were not agreeable. Parents or guardians of eligible children were contacted by the investigator on the day prior to the operation and written informed consent from a parent and/or legal guardian for study participation and assent for those aged > 7 years were obtained.

### Standard operating procedure

At the operating theater, the standard ASA monitoring (noninvasive blood pressure, electrocardiography, pulse oximetry, and end-tidal CO_2_ concentration) was applied to all patients. Each patient was placed in the standard sniffing position. The standard protocol of propofol 1–5 mg kg^−1^, fentanyl 1–2 mcg kg^−1^, and cisatracurium 0.15 mg kg^−1^ was given. Neuromuscular monitoring [TOF-watch® SX (Organon Ltd., Dublin, Ireland)] was applied after induction to ensure neuromuscular blockage for ultrasonography performance and intubation. Ultrasonography of subglottic diameter and glottic transverse diameter was performed by two investigators during face mask ventilation when the train of four (TOF) showed 4 or lower. Tracheal intubation was allowed after the TOF count was showed 0, or the patient was ventilated for more than 5 min. The uncuffed ETT (Shiley™) size was chosen on the discretion of the attending anesthesiologist. A leak test was done after successful intubation. An adjustable pressure-limiting (APL) valve was set at 15–25 cm H_2_O and the anesthesiologist staff used a stethoscope to detect leakage at the mid trachea. Management of improper uncuffed ETT size depended on the attending anesthesiologist. We used the optimal air leak test at an airway pressure between 15 and 25 cm H_2_O to avoid smaller-sized uncuffed ETT. If the first attempt succeeded but the leak test was less than 15 cm H_2_O, a 0.5 mm ID larger uncuffed ETT or cuffed ETT was considered for the second attempt to satisfy the leak test. If the air leak was not observed between the airway pressure of 15 and 25 cm H_2_O, the airway pressure would be increased until air leak was observed. If an air leak pressure of more than 25 cm H_2_O was observed after successful intubation, the ETT size was changed to a 0.5 mm ID smaller uncuffed ETT size depending on the discretion of the attending anesthesiologist.

### Neck ultrasonography to measure glottic transverse diameter and subglottic diameter

**Figure 1:**
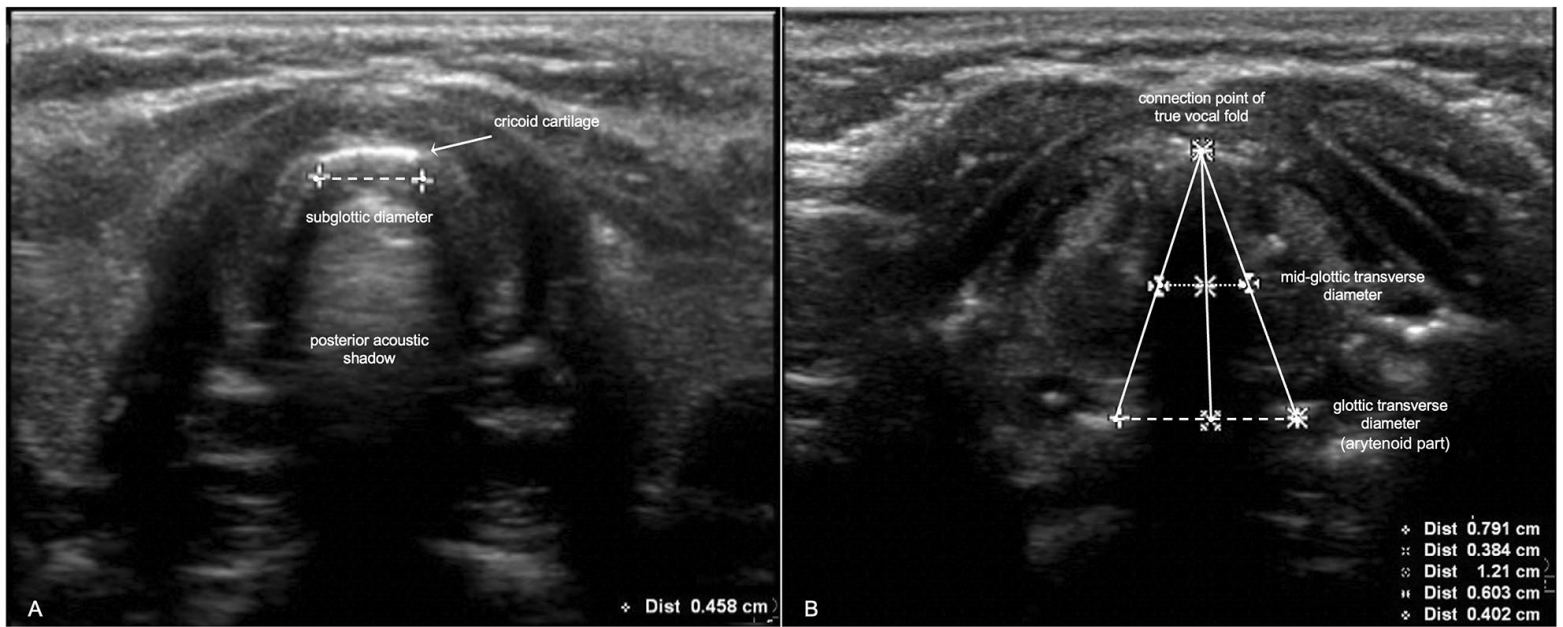
Subglottic diameter measurement (**A**), and glottic/mid-glottic transverse diameter measurement (**B**).

An ultrasound linear transducer (Philips—Affinity 50, Philips, Inc.) of 12–4 MHz or 12–5 MHz was used to measure the subglottic diameter and glottic transverse diameter by two investigators (CD, MO). The technique of ultrasonography was begun to identify the hyoid bone, which appeared at the superficial, hyperechoic curvilinear structure with posterior acoustic shadowing in the transverse view. Then, the probe was moved caudally to localize the glottic transverse diameter by anatomical landmark at the thyroid cartilage. Vocal muscles were identified bilaterally as false vocal fold and true vocal fold bilaterally. Then, the probe was moved caudally to visualize the cricoid arch. The cricoid cartilage was located above the air-mucosa interface (or air-column) which was the hyperechoic part. The subglottic diameter was identified at this level^8,14^. The subglottic diameter was measured between the bilateral margins of the mid cricoid cartilage beyond the air-column (Fig. 1A)^8,14^. The process of measuring the mid-glottic transverse diameter began by identifying both arytenoids. The true vocal cords were abducted, which was considered the connection point of the true vocal fold as the top of the true vocal fold triangle where the distance between arytenoids was considered as the glottic transverse diameter (arytenoid part or base of triangle). The mid-glottic transverse diameter was measured by forming an imaginary transverse line between the half distance of both arytenoids and connection point of the true vocal fold^11^ (Fig. 1B). The video recording was performed during ultrasound performance and was reviewed by the investigator (CD) to measure the glottic transverse and subglottic diameters.

### Inter-rater and intra-rater variability

We recruited 20 patients in a pilot study to measure the subglottic and glottic transverse diameter by two investigators (CD, MO). The intra-rater correlations of the subglottic diameter were 0.989 and 0.984 while for the glottic transverse diameter were 0.962 and 0.773, respectively. The inter-rater correlation of the subglottic diameter was 0.859 while that of the glottic transverse diameter was 0.902.

### Outcome measurement

The final internal diameter (ID) of uncuffed ETT was recorded for each patient and was converted to OD (mm) based on Shiley™ ETT conversion table to represent the outcome variable. For patients who were successfully intubated with a cuffed ETT, 0.5 mm. ID was added to the final size and was converted to OD (mm) in the same manner.

### Sample size determination

The sample size was calculated based on the correlation between the subglottic diameter and OD of ETT size. A correlation coefficient (r) of 0.3, which represented a medium level of correlation, was used to calculate a required sample size of 85 patients under a significance level of 0.05. With the assumption that 10% of study participants would withdraw from the study, we increased the sample size to 95.

### Statistical analysis

A data record form was created and the data were double-entered into a database using EpiData version 3.1. R version 4.1.1 was used for all analysis (R Core Team, Vienna). Categorical variables were reported as frequency and percentage while continuous variables were presented as mean and standard deviation (SD) or median and interquartile range (IQR) where appropriate. Pearson's correlation coefficients (r) were used to determine the degree of correlation between subglottic diameter, mid-glottic transverse diameter, glottic transverse diameter and OD of ETT. Linear regression was used to determine the optimum formula to predict the OD of ETT. The mean differences and 95% confidence interval (CI) between predicted ETT size calculated from the formulae of subglottic diameter and mid-glottic transverse diameter and the formulae of subglottic diameter and glottic transverse diameter were assessed using Bland and Altman plots.

### Ethics approval and consent to participate

The study was approved by the Institutional Ethics Committee of the Faculty of Medicine, Prince of Songkla University, Songkhla, Thailand, Chairperson Assoc. Prof. Boonsin Tangtrakulwanich, EC #6213384 on June 10, 2019. The written inform consent to participate was obtained.

## Results

**Figure 2:**
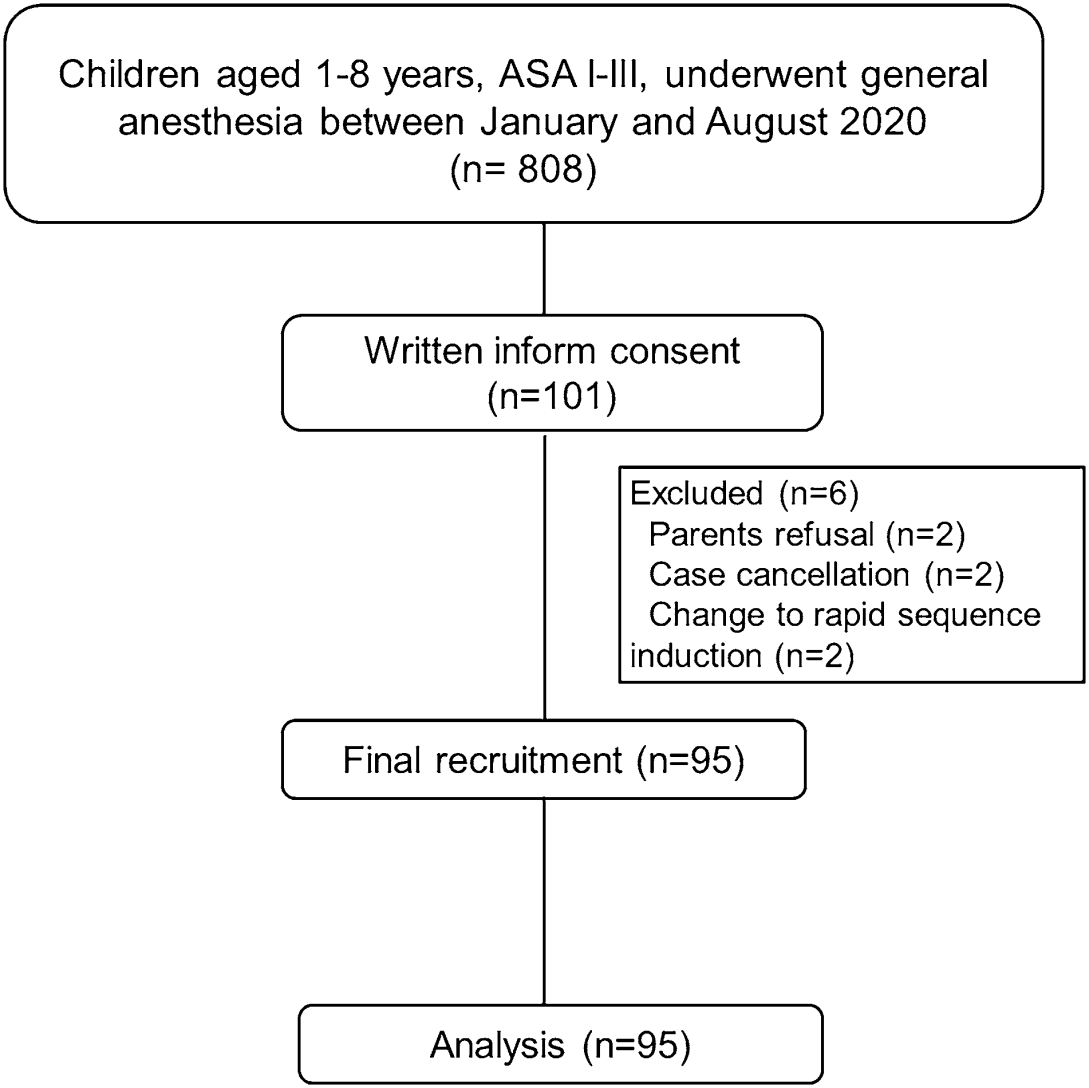
Flow diagram of the study. *ASA* American Society of Anesthesiologists.

One hundred and one children aged between 1 and 8 years were assessed for eligibility, of which 6 were excluded before their operation (Fig. 2). Therefore, ninety-five children were enrolled. The demographic data, surgery and anesthesia related data is shown in Table 1. The mean (SD) age of all study participants was 4.57 (2.06) years. About half had ASA classification I. Anemia (18.9%) and genetic disorders (7.4%), which did not correlate with major airway abnormality, were the most common coexisting diseases. Eye surgery was the most common type of surgery (64%). Before intubation, 62% of participants had a TOF count of 0. The mean (SD) duration of the two ultrasonography performances was 3.40 (1.14) minutes. Fifty-two patients (55%) required a second or third attempt, of which 27 patients (29%) required a cuffed ETT.

**Table 1:** Demographic data, surgery and anesthesia related data (N = 95).

Variable	N (%) or mean ± SD
Age (years)	4.57 ± 2.06
Male	55 (57.9)
Body weight (kg)	17.10 ± 6.01
Height (cm)	102.29 ± 14.84
ASA classification	
I	47 (49.5)
II	39 (41.1)
III	9 (9.5)
Coexisting diseases	
Upper respiratory tract infection	3 (3.2)
Anemia	18 (18.9)
Genetic disorder	7 (7.4)
Cardiovascular system disorder	2 (2.1)
Respiratory disorder	7 (7.4)
Central nervous system disorder	4 (4.2)
Obesity (> 95th percentile weight)	7 (7.4)
Type of surgery	
Eye	61 (64.2)
Thoracic surgery	3 (3.2)
Orthopedics	9 (9.5)
Urologic surgery	1 (1.1)
Plastic surgery	6 (6.3)
General surgery	12 (12.6)
Ear-nose-throat	3 (3.2)
Duration of ultrasound (seconds)	204.4 ± 68.6
Intubation attempts	
1	43 (45.3)
2	48 (50.5)
3	4 (4.2)

Data presented as frequency (%) and mean ± standard deviation.

*ASA* American Society of Anesthesiologists.

### Correlation between glottic transverse diameter/subglottic diameter and ETT size

**Figure 3:**
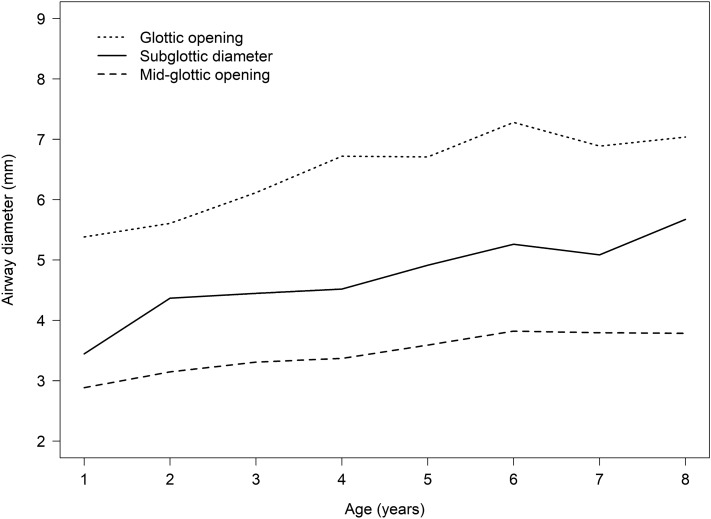
Relationship between airway ultrasound diameter and age.

Measurements of subglottic diameter, mid-glottic transverse diameter, and glottic transverse diameter by ultrasonography are shown in Table 2. The relationship between airway ultrasound diameter and age is shown in Fig. 3. The airways were smallest in the mid-glottic transverse region and largest in the glottic transverse region compared to subglottic region which was consistent throughout all ages.

**Table 2:** Measurement of subglottic diameter, mid-glottic and glottic transverse diameter at the age of 1–8 years (N = 95).

Age (years)	Subglottic diameter (mm)	Mid-glottic transverse diameter (mm)	Glottic transverse diameter (mm)	Outer ETT diameter (mm)	ETT size (mm ID)
1 (n = 7)	3.5 [3.1, 3.5]	2.8 [2.7, 2.9]	5.2 [4.9, 5.6]	6.2 [6.2, 6.2]	4.5 [4.5, 4.5]
2 (n = 20)	4.1 [3.6, 5.0]	3.0 [2.6, 3.4]	5.4 [5.0, 6.3]	6.2 [6.2, 6.8]	4.5 [4.5, 5.0]
3 (n = 14)	4.5 [4.2, 4.7]	3.4 [2.9, 3.6]	6.3 [5.1, 6.7]	6.8 [6.8, 7.5]	5.0 [5.0, 5.5]
4 (n = 12)	4.4 [4.0, 4.8]	3.3 [3.0, 3.5]	6.4 [5.7, 6.9]	7.5 [6.8, 7.5]	5.5 [5.0, 5.5]
5 (n = 15)	4.6 [4.3, 5.0]	3.5 [3.3, 4.0]	6.5 [6.0, 7.6]	7.5 [7.5, 8.2]	5.5 [5.5, 6.0]
6 (n = 14)	4.9 [4.5, 5.9]	3.6 [3.4, 3.7]	6.9 [6.5, 7.4]	7.8 [7.5, 8.2]	6.0 [5.5, 6.0]
7 (n = 6)	5.1 [4.8, 5.2]	3.6 [3.2, 4.4]	6.8 [6.3, 7.7]	7.8 [7.5, 8.2]	6.0 [5.5, 6.0]
8 (n = 7)	5.8 [5.2, 6.4]	3.9 [3.6, 4.0]	6.6 [6.4, 7.5]	8.8 [8.2, 8.8]	6.5 [6.0, 6.5]

Data presented as median [interquartile range].

*ETT* endotracheal tube.

**Figure 4:**
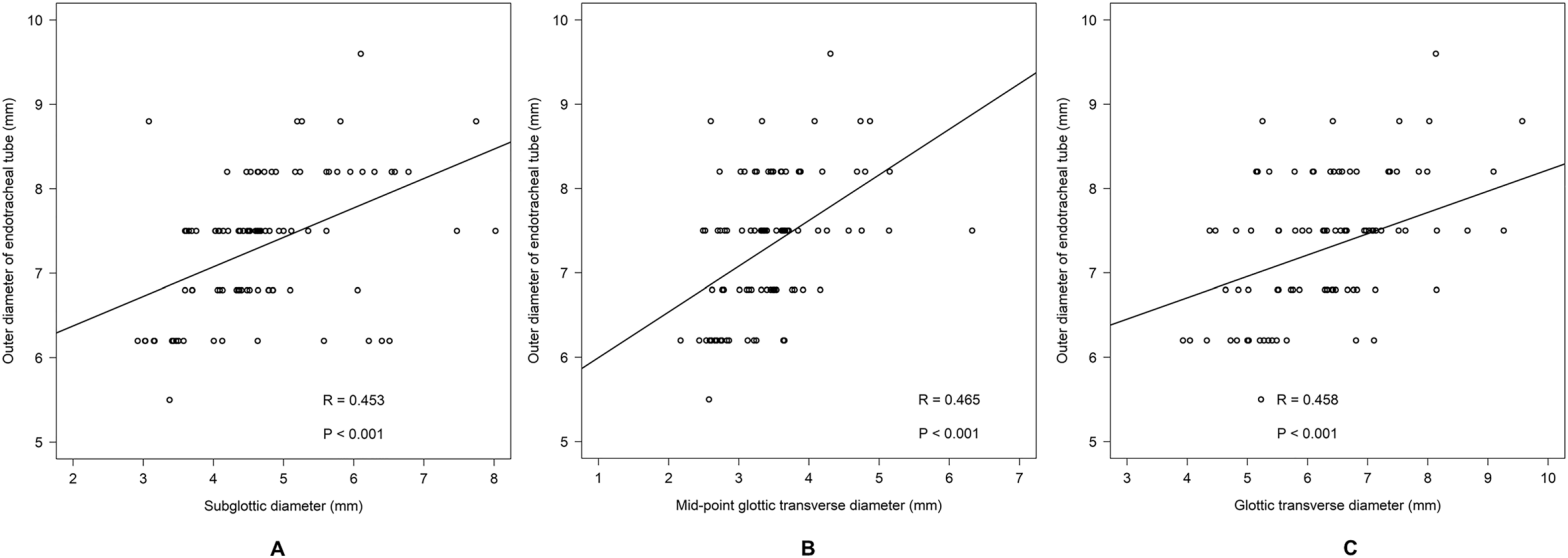
Scatter plots showing the relationships between subglottic diameter and endotracheal tube size (**A**), between mid-glottic transverse diameter and endotracheal tube size (**B**), and between glottic transverse diameter and endotracheal tube size (**C**).

**Figure 5:**
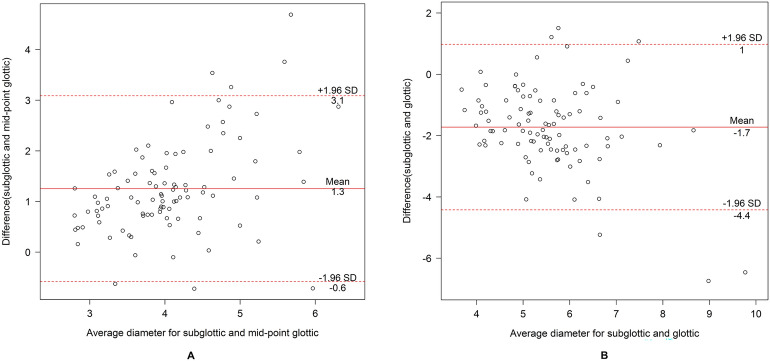
Bland–Altman plots of subglottic diameter-mid-glottic diameter (**A**) and subglottic diameter-glottic diameter (**B**).

Table 3 shows the formulae of subglottic diameter, mid-glottic transverse diameter, and glottic transverse diameter to predict the OD of ETT and the correlations among them. The correlation between glottic transverse diameter and mid-glottic transverse diameter was high (r = 0.9) whereas that between subglottic diameter and mid-glottic transverse diameter was moderate (r = 0.5). Figure 4 shows scatter plots between OD of ETT (mm) and subglottic diameter (4A), mid-glottic transverse diameter (4B), and glottic transverse diameter (4C). The overall median [IQR] of the diameter differences between the subglottic diameter and mid-glottic transverse diameter was 1.1 [0.7, 1.6] mm. The Bland–Altman plot of subglottic diameter and mid-glottic transverse diameter showed a mean difference of + 1.3 mm (95% CI of + 3.1 and − 0.6) (Fig. 5A) and that of subglottic diameter and glottic transverse diameter was − 1.7 mm (95% CI of + 1.0 and − 4.4) (Fig. 5B).

**Table 3:** Formulae of subglottic diameter, mid-glottic and glottic transverse diameter to predict outer diameter of endotracheal tube size and the correlations among them (N = 95).

Formula to predict outer diameter	R (95% CI)	*p*-value*
5.7 + (subglottic_mm_/3)	0.45 (0.28, 0.60)	< 0.001
5.5 + (mid-glottic_mm_/2)	0.47 (0.29, 0.61)	< 0.001
5.7 + (glottic_mm_/4)	0.46 (0.28, 0.60)	< 0.001

Correlations	R (95% CI)	Difference (mm)**
Subglottic and mid-glottic diameter	0.50 (0.34, 0.64)	1.09 [0.72, 1.60]
Glottic and subglottic diameter	0.46 (0.28, 0.60)	1.82 [0.86, 2.34]
Glottic and mid-glottic diameter	0.89 (0.84, 0.93)	2.93 [2.45, 3.39]

*CI* confidence interval.

*Pearson's correlation.

**Median [interquartile range].

## Discussion

We found a moderate correlation among ultrasonography measurements of the glottic region (r value of 0.46 for glottic transverse diameter and 0.47 for mid-glottic transverse diameter) and subglottic diameter (r value of 0.45) to predict OD of uncuffed ETT size in children. Since this was the first study to examine glottic region by ultrasonography to predict ETT size in children, we examined two measurements of the glottic region; glottic transverse diameter and mid-glottic transverse diameter. Since the shape of a true vocal fold is somewhat triangular with both arytenoids considered as the base of the triangle (Fig. 1B), calculation of ETT size based on the glottic transverse diameter might result in an ETT size that is too large. Therefore, the mid-glottic transverse diameter was also examined. To determine the fitted uncuffed ETT size, we used a leak pressure of 15–25 cm H_2_O to ensure optimal ETT size, which was consistent with the study of Makireddy^15^.

Since there is evidence that the subglottic transverse diameter is less than the anterior to posterior (A-P) diameter^16,17^, we measured the subglottic transverse diameter, which we believed to be the narrowest part of the child's airway and consistent with other studies^6–9^. The correlations between the subglottic diameter and outer diameter of ETT in our study were moderate whereas other studies recruiting children aged ≤ 6 years found a strong correlation between the subglottic diameter and outer diameter^6,8,18^. Moreover, we measured the narrowest part just below the cricoid ring (Fig. 1A) which was more anteriorly close to the cricoid ring compared to other studies which were measured more caudally. Therefore, our measurement of subglottic diameter, which we believe to be closer to the narrowest part, might be slightly smaller than measurements from other studies^19–25^. The correlation between the glottic/mid-glottic transverse diameter and ETT size was not different compared to the correlation between subglottic diameter and ETT size. However, ultrasonography of the subglottic region may be more assessable or easier than the glottic region due to the small airway, especially in the new performers.

Previous studies in children reported that the narrowest part of the larynx was the transverse dimension at the level of the vocal cord (as opposed to the cricoid ring) by MRI^10^ and rigid video-bronchoscopy study^11^. Since the glottic opening dimension are dependent on the extent of muscle paralysis, children undergoing MRI and video bronchoscopy are usually spontaneously breathing and will have smaller distances than paralyzed children. From our results which using neuromuscular blocking agent, the glottic transverse diameter was larger than the subglottic diameter and the smallest airway was the mid-glottic transverse diameter. Therefore, the glottic region could be the narrowest part if it was measured at the mid-glottic level compared to the glottic level (base of true vocal fold triangle).

Husein et al^26^ found that the diameters of videobronchoscopy measurement, ETT size, and subglottic diameter by ultrasonography ranged from the largest to the smallest measurement, respectively. This supported our results in which the mid-glottic and subglottic measurements, and not the glottic transverse measurement, were smaller than the actual ETT size. Therefore, using different formulae in airway ultrasonography for calculation of ETT size in children are useful if the ultrasound performer can access only the glottic or subglottic regions. By ultrasonography measurement, we found that the subglottic diameter was 1.1 mm larger than the mid-glottic transverse diameter. However, according to the Bland and Altman plots, there were no significant differences in transverse diameter between subglottic and mid-glottic transverse diameter to predict ETT size (mean of + 1.3 mm). Therefore, the glottis/mid-glottic transverse diameter formulae can be used to predict ETT size as well as the well-known subglottic diameter measurement formula.

The applications of the study are as follow. We found a large inter-individual variability in glottic and subglottic internal airway dimensions for a specific age, which supports the use of cuffed ETT in children at least 1 year of age. However, in our institute, the decision to use either a uncuffed or cuffed ETT is based on the individual judgement of the anesthesiologist in charge. Both the subglottic diameter and glottic transverse diameter formulae can be used to predict uncuffed or cuffed ETT size. For predicting the cuffed ETT size, the 0.5 mm ID smaller size of subglottic/glottic formulae can be applied. Moreover, the glottic transverse diameter, which is the distance between both sides of the arytenoids, is simpler to measure than the mid-glottic transverse diameter. In case of a difficult subglottic approach, the glottic region approach could be used instead. However, an experienced ultrasound pediatric anesthesiologist is required for measuring this diameter in children with small airways. Further studies to find the agreement between subglottic and glottic transverse diameter formulae and actual ETT size should be performed.

Some strengths of this study are as follows. First, we used the average measurement of subglottic/glottic transverse diameter ultrasonography of the two investigators to provide high inter-rater reliability and intra-rater reliability and avoid performance bias from a single operator. Second, a neuromuscular function monitor was used to ensure the full abduction phase or maximum transverse diameter of the glottis transverse diameter during ultrasonography performance. Despite these strengths, our study has some limitations. First, since patients of various ages were included, the older aged (> 6 years) children could pass the air leak test with a cuffed ETT. Thus, the calculation of uncuffed ETT size might not be accurate for the smaller cuffed ETT. Second, according to the study design, our subjects may not have benefited from receiving less chance of ETT size changing from the ultrasonography performance. The subglottic/glottic transverse diameter formulae to estimate the ETT size were provided after the process of data analysis. However, only 4% of our subjects required a third attempt. Finally, generalizability of our study is confined to only healthy children with no previous airway abnormalities.

## Conclusion

Based on ultrasonography, a moderate correlation between the glottic transverse diameter and subglottic diameter was found for predicting ETT size in children. The ultrasound measurement of the glottic transverse diameter can be used with the subglottic diameter to predict ETT size or can be used instead of the subglottic diameter if a difficult approach is encountered.

## Supplementary Information

Supplementary Information.

## Data Availability

All data generated or analysed during this study are included in the supplementary files.
